# Cell Surface Proteins for Enrichment and *In Vitro* Characterization of Human Pluripotent Stem Cell-Derived Myogenic Progenitors

**DOI:** 10.1155/2022/2735414

**Published:** 2022-02-24

**Authors:** Sin-Ruow Tey, Madison Mueller, Megan Reilly, Colton Switalski, Samantha Robertson, Mariko Sakanaka-Yokoyama, Masatoshi Suzuki

**Affiliations:** ^1^Department of Comparative Biosciences, University of Wisconsin-Madison, Wisconsin, USA; ^2^Stem Cell and Regenerative Medicine Center, University of Wisconsin-Madison, Wisconsin, USA

## Abstract

Human myogenic progenitors can be derived from pluripotent stem cells (PSCs) for use in modeling natural and pathological myogenesis, as well as treating muscle diseases. Transgene-free methods of deriving myogenic progenitors from different PSC lines often produce mixed populations that are heterogeneous in myogenic differentiation potential, yet detailed and accurate characterization of human PSC-derived myogenic progenitors remains elusive in the field. The isolation and purification of human PSC-derived myogenic progenitors is thus an important methodological consideration when we investigate the properties and behaviors of these cells in culture. We previously reported a transgene-free, serum-free floating sphere culture method for the derivation of myogenic progenitors from human PSCs. In this study, we first performed comprehensive cell surface protein profiling of the sphere culture cells through the screening of 255 antibodies. Next, we used magnetic activated cell sorting and enriched the cells according to the expression of specific surface markers. The ability of muscle differentiation in the resulting cells was characterized by immunofluorescent labeling and quantification of positively stained cells. Our results revealed that myotube-forming cells resided in the differentiated cultures of CD29^+^, CD56^+^, CD271^+^, and CD15^–^ fractions, while thick and multinucleated myotubes were identified in the differentiated cultures from CD9^+^ and CD146^+^ fractions. We found that PAX7 localization to the nucleus correlates with myotube-forming ability in these sorted populations. We also demonstrated that cells in unsorted, CD271^+^, and CD15^–^ fractions responded differently to cryopreservation and prolonged culture expansion. Lastly, we showed that CD271 expression is essential for terminal differentiation of human PSC-derived myogenic progenitors. Taken together, these cell surface proteins are not only useful markers to identify unique cellular populations in human PSC-derived myogenic progenitors but also functionally important molecules that can provide valuable insight into human myogenesis.

## 1. Introduction

Myogenic progenitors, also known as skeletal muscle progenitor/stem cells, can differentiate into skeletal myocytes and form contractile muscle units required for muscle repair and regeneration. Various sources have been used to propagate myogenic progenitors in culture, including fetal muscle, adult muscle, and nonmuscle somatic tissues [1–5]. However, muscle biopsy-derived adult myogenic progenitors are expandable with limited passage numbers and rapidly undergo senescence in culture. These issues are being resolved with the advancement of stem cell technology. Specifically, human pluripotent stem cells (PSCs), which include embryonic stem cells (ESCs) and induced pluripotent stem cells (iPSCs), are a virtually indefinite new cell source for efficient and cost-effective myogenic progenitor preparation [6–8]. Human PSC-derived myogenic progenitors can provide valuable insights into the mechanisms of natural and pathological myogenesis via *in vitro* modeling and *in vivo* experimentation. For instance, *in vitro* drug screening using human PSC-derived myogenic progenitors allows us to identify new mechanisms and molecules that have the capacity to prevent muscle wasting and atrophy during normal aging or disease processes. In the last decade, several approaches were proposed from different laboratories for efficient derivation of myogenic progenitors from human PSCs. In particular, a series of our published studies demonstrated the feasibility of producing myogenic progenitors from human PSCs directly without genetic modification using serum-free and feeder-free floating spherical culture (EZ spheres) [6, 9, 10].

Specific cell surface proteins can be used to isolate, identify, and characterize viable human myogenic progenitors [11]. As human PSC derivatives often display varying heterogeneity in cell types, enrichment using cell surface markers is an essential step in current procedures to improve the purity of the myogenic progenitor population. Although markers of rodent satellite cells have been extensively studied, data on human myogenic progenitor markers is limited—only specific transcriptional factors (such as PAX3, PAX7, Myf5, and MyoD) and cell surface markers (CD29 and CD56) have been widely accepted as reliable early human satellite cell markers [11]. Among them, transcription factors are incompatible for live cell isolation because of their nuclear localization. Since CD29 expression was also observed on some nonmuscle cells within muscle tissue [12], CD29 alone is ineffective for the identification of human myogenic progenitors and has never been used as a sole marker to isolate myogenic progenitors prepared from human PSCs [11]. CD56 can be used as a sole marker to isolate myogenic progenitors derived from adult muscle [13–16], but its specificity and efficiency as a sole marker for isolation of PSC-derived myogenic progenitors remain unknown [11]. It has also been reported that cells lacking CD56 expression can exhibit myogenic progenitor properties [17, 18]. Moreover, variations of derivation methods and PSC lines differently produce mixed cell populations. To date, there is no consensus on a common sole marker or a gold standard combination of multiple markers for purification of human PSC-derived myogenic progenitors in various settings. For example, the combination of myogenic progenitor markers CD271 and ErbB3 yielded contradictory results when used to isolate human PSC-derived myogenic progenitors prepared via different protocols [19, 20].

To better characterize and enrich human PSC-derived myogenic progenitors, we performed comprehensive profiling of cell surface markers using EZ sphere cells by screening with 255 antibodies. Based on expression of selected markers, we then sorted EZ sphere cells using magnetic activated cell sorting (MACS). Compared to fluorescence-activated cell sorting (FACS), MACS processing results in about 10 times higher cell viability [21] and higher postsort population growth [22]. For a single sample, MACS processing is 4-6 times faster than FACS; for multiple samples, MACS can be performed in parallel while FACS needs to be performed serially. Furthermore, all sorting procedures of MACS can be completed within a biosafety cabinet, which is easily available for a wide range of researchers without an expensive cell sorter. The sorted cells were differentiated to evaluate their myogenic potential using immunocytochemical analysis. We found that cells with improved myotube-forming efficiency resided in the differentiated cells of the CD29^+^ fraction, CD56^+^ fraction, CD271^+^ fraction, and CD15^–^ fraction, whereas the differentiated cultures of the CD9^+^ fraction and CD146^+^ fraction showed improved myotube fusion. Detailed analysis of Pax7 intracellular distribution revealed higher occurrence of Pax7 localization into cell nuclei among the undifferentiated cultures of the CD9^+^ fraction, CD29^+^ fraction, CD56^+^ fraction, CD271^+^ fraction, and CD15^–^ fraction, suggesting a positive correlation between nuclear Pax7 expression and myotube-forming ability. Furthermore, undifferentiated cells of the CD271^+^ fraction and CD15^–^ fraction exhibited improvement in the expansion rate compared to unsorted populations and retained myotube-forming efficiency upon induction of terminal differentiation. Lastly, we observed that inhibition of CD271 expression caused impairment in myogenic differentiation. Our findings implied that these cell surface proteins may be functionally essential molecules that can unveil important information about human muscle biology and diseases.

## 2. Materials and Methods

### 2.1. Human Pluripotent Stem Cells

The human ESC line WA09 (H9) and human iPSC line IMR-90 were obtained from WiCell (Madison, WI, USA). These lines were maintained using a feeder-free protocol [23]. iPSC colonies were cultured in mTeSR1 (WiCell) medium on a 6-well plate coated with Matrigel (BD Bioscience; San Jose, CA) and passaged using Versene (Life Technologies, Grand Island, NY, USA).

### 2.2. Differentiation of iPSCs to Myogenic Progenitors and Myotubes

Human iPSC-derived myogenic progenitors and myotubes were prepared using our protocol as recently described [6]. Briefly, iPSC colonies were manually lifted using a scraper and transferred into expansion medium consisting of Stemline medium (S-3194, Sigma-Aldrich, St. Louis, MO, USA), 100 ng/ml human fibroblast growth factor-2 (FGF-2; WiCell), 100 ng/ml human epidermal growth factor (EGF; Millipore, Billerica, MA, USA), 5 ng/ml heparin sulfate (Sigma-Aldrich), and penicillin/streptomycin/amphotericin B (PSA, 1%*v*/*v*; Life Technologies). For a negative control, iPSC colonies were transferred into the expansion medium without FGF-2. The colonies were maintained in the culture flask precoated with poly (2-hydroxyethyl methacrylate) (poly-HEMA; Sigma-Aldrich) to prevent cell attachment to the surface. After 1 week, the colonies formed free-floating spherical aggregates as EZ spheres. The spheres were passaged weekly by mechanical chopping using a McIlwain tissue chopper (Mickle Laboratory Engineering, Surrey, UK). Mechanical chopping allows the maintenance of cell-to-cell contact of spherical cultures for rapid and stable expansion without dissociation to a single cell suspension [24–26]. The tissue chopper was sterilized by spraying with 70% isopropanol and operated inside a biosafety cabinet. A flame-sterilized razor blade was set onto the arm of the tissue chopper. Spheres were placed on a petri dish lid and mechanically chopped into 200 *μ*m cubes.

After 6 weeks, EZ spheres were dissociated using trypsin (TrypLE, Life Technologies), triturated in terminal differentiation medium (Dulbecco's Modified Eagle's Medium (DMEM, Sigma-Aldrich) containing 2% B27 serum-free supplement (Life Technologies) and 1% PSA), passed through a cell strainer with 40 *μ*m mesh pore size (352340, Corning Inc., Corning, NY, USA), and plated at a density of 4000 cells/*μ*l on glass coverslips coated with poly-L-lysine (0.1 mg/ml) and laminin (50 *μ*g/ml) (both Sigma-Aldrich) [6]. The plated cells were then maintained in 24-well plates for 2 weeks in terminal differentiation medium for differentiation into myotubes.

### 2.3. Surface Marker Screening

Screening was performed with the BD Lyoplate™ Human Cell Surface Marker Screening Panel (BD Biosciences), which contains 242 purified monoclonal antibodies against human cell surface markers and corresponding secondary antibodies. Furthermore, the following primary antibodies against cell surface markers were also added to the screening: CD331, CD332, CD333, and CD334 (Cell Signaling Technology, Danvers, MA, USA). These four surface markers are members of the fibroblast growth factor receptor (FGFR) family expressed on satellite cells and involved in myogenesis, also known as FGFR1, FGFR2, FGFR3, and FGFR4, respectively [27, 28]. iPSC colonies were transferred into expansion medium as described above. EZ spheres were maintained as free-floating spherical culture and passaged through mechanical chopping once a week for 5 weeks, then dissociated and triturated in the terminal differentiation medium, and passed through a cell strainer with 40 *μ*m mesh pore size as described above. 1.5 × 10^4^ cells per well were plated in 96-well glass round-bottom plates coated with poly-L-lysine (0.1 mg/ml) and laminin (50 *μ*g/ml) (both Sigma-Aldrich). Plated cells were maintained in the terminal differentiation medium overnight. Next, cells were incubated with primary antibodies (25 *μ*g/ml) on ice for 30 minutes and after phosphate-buffered saline (PBS) wash incubated with secondary antibodies (1.25 *μ*g/ml) on ice for 30 minutes. After another PBS wash, cells were fixed with ice-cold methanol for 20 minutes and then incubated with Hoechst 33258 (0.5 *μ*g/ml in PBS; Sigma-Aldrich) for 10 minutes. Images were acquired by a Nikon Eclipse TE2000-E with a DS-QilMC CCD camera (Nikon, Tokyo, Japan) and a Zeiss META 510 laser scanning confocal microscope (Zeiss, Jena, Germany).

### 2.4. Magnetic Activated Cell Sorting (MACS)

**Figure 1:**
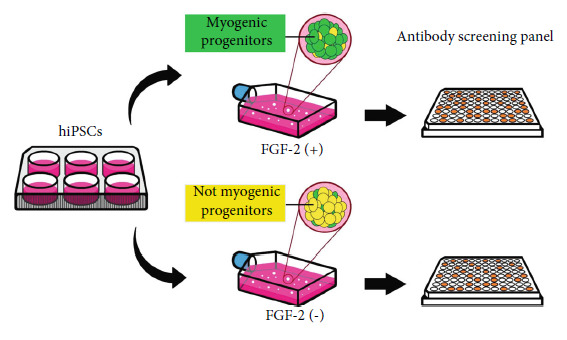
Graphical abstract of antibody screening for surface marker profiling of human PSC-derived myogenic progenitors. FGF-2-treated and nontreated cells derived from human PSCs were stained with 255 antibodies against surface markers using the BD Lyoplate™ Human Cell Surface Marker Screening Panel.

**Figure 2:**
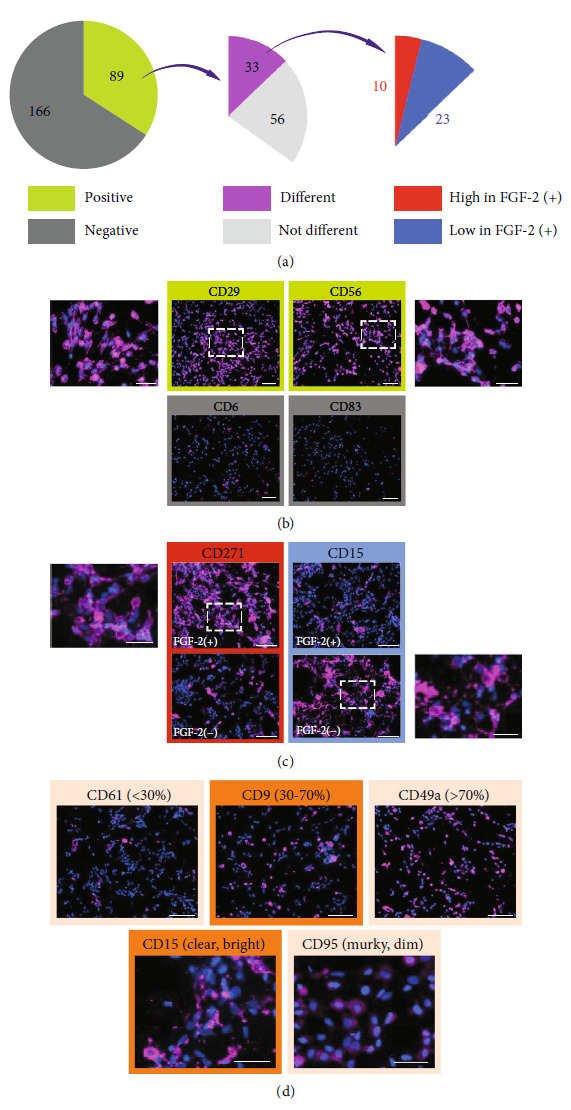
Selection of potential surface markers to enrich human PSC-derived myogenic progenitors. (a) 33 antibodies among the 89 antibodies stained positive in FGF-2-treated cells showed noticeable different expression levels in nontreated cells: 10 showed higher expression in FGF-2-treated cells and 23 showed lower expression in FGF-2-treated cells. (b) FGF-2-treated cells stained positive for CD29 and CD56 but negative for CD6 and CD83. Pink = positive for the surface marker indicated; blue = all nuclei. Scale bar = 100 *μ*m; enlarged image scale bar = 50 *μ*m. (c) Higher CD271 expression was seen in FGF-2 treated cells, whereas CD15 expression was higher in nontreated cells. Scale bar = 100 *μ*m; enlarged image scale bar = 20 *μ*m. (d) CD9 and CD15 fit our criteria of selection that 30-70% of myogenic progenitor culture was stained positive with high intensity and clear morphology; CD61, CD49a, and CD95 did not fit the criteria. Top panel scale bar = 100 *μ*m; bottom panel scale bar = 50 *μ*m.

iPSC-derived EZ spheres were cultured in expansion medium for at least 6 weeks and used for cell sorting using Miltenyi Biotec (Bergisch Gladbach, Germany) column-based sorting kit. EZ spheres were dissociated, triturated, and passed through a cell strainer as described above. Some cells were incubated with magnetic bead-conjugated antibodies (1 : 5 dilution in MACS buffer solution, used 100 *μ*l per 10^7^ total cells) at 4°C for 15 minutes. Magnetic bead-conjugated antibodies used were CD15-MicroBeads and CD56-MicroBeads (Miltenyi Biotec). Other cells were incubated with fluorochrome-conjugated antibodies against surface markers of interest (1 : 10 dilution in MACS buffer solution, used 100 *μ*l per 10^7^ total cells) at 4°C for 15 minutes, washed with MACS buffer (5% MACS BSA Stock Solution in autoMACS Rinsing Solution™; Miltenyi Biotec), and incubated with MicroBead-conjugated antibodies against appropriate fluorochromes (10 *μ*l antibodies in 90 *μ*l MACS buffer solution per 10^7^ total cells) at 4°C for 10 minutes. Fluorochrome-conjugated antibodies used were CD9-fluorescein isothiocyanate (FITC), CD26-FITC, CD49b-FITC, CD49f-FITC, CD98-FITC, CD146-FITC, CD164-FITC (BD Biosciences), CD13-FITC, CD24-FITC, CD29-FITC, CD82-FITC, CD184-allophycocyanin (APC), CD271-phycoerythrin (PE), ErbB3-FITC (Miltenyi Biotec), and ITGN-*α*7-FITC (Biorbyt, Cambridge, UK). These antibodies were selected based on the screening described above and on Figures 1 and 2. Microbead-conjugated antibodies used were anti-FITC microbeads, anti-APC microbeads, and anti-PE microbeads (Miltenyi Biotec). The details of these surface marker antibodies are available in Supplementary Table S1.

Labelled cells were then passed through the magnetic columns set up in MACS Manual Separators for sorting, as described by the manufacturer (Miltenyi Biotec). Their MS and LD columns were used for positive and negative selections, respectively. Sorted cells were plated down on coverslips and maintained in terminal differentiation medium for two weeks and then fixed with methanol and analyzed. For each experiment, a parallelly prepared group of cells that did not go through MACS was used as control, referred to as “unsorted cells.”

### 2.5. Immunocytochemistry

Cells were processed for PAX7 and myosin heavy chain (MyHC) immunocytochemistry as described previously [29]. For PAX7 staining, plated cells on coverslips were fixed with 4% paraformaldehyde (PFA) in PBS; for MyHC staining, plated cells on coverslips were fixed with ice-cold methanol. Fixed cells were permeabilized with 0.1% Triton X-100 and 5% normal donkey serum (NDS) in PBS. After rinsing with PBS, the cells were incubated with primary antibodies against PAX7 (1 : 40; Developmental Studies Hybridoma Bank, DSHB, Iowa City, IA) or MyHC (MF20, 1 : 20; DSHB). After incubation with the primary antibodies, the cells were rinsed with PBS and incubated with a secondary antibody conjugated with Alexa Fluor 488 or Cy3 (1 : 1,000; Jackson ImmunoResearch Laboratories, West Grove, PA). Lastly, cell nuclei were stained with Hoechst 33258 as described above. Coverslips were mounted on slides using a mounting medium (Fluoromount-G; SouthernBiotech, Birmingham, AL, USA).

For surface marker immunocytochemistry, live cells were plated on coverslips and then incubated with FITC-conjugated antibodies against CD9, CD15, CD26, CD29, CD49b, CD49f, CD56, CD98, CD146, CD164, CD271 (BD Biosciences), CD184 (BioLegend, San Diego, CA, USA), CD13, CD24, CD82, ERBB3 (Miltenyi Biotec), and ITGN-*α*7 (Biorbyt) or with unconjugated primary antibodies against CD55, EGFR (BD Biosciences), CD34 (Invitrogen), CD331, CD332, CD333, and CD334 (Cell Signaling Technology), with the dilution to 1 : 40 for an hour at room temperature. A list of primary antibodies is available in Supplementary Table S1. When using unconjugated primary antibodies, the stained cells were rinsed with PBS and incubated with a secondary antibody conjugated to Alexa Fluor 488 with the dilution to 1 : 1,000 for 30 minutes at room temperature in the dark. The cells incubated with FITC-conjugated primary antibodies skipped the secondary antibody incubation and proceeded to the next step. After rinsing with PBS, cells were fixed and stained with Hoechst 33258 as described above. Coverslips were then mounted on slides using a mounting medium (Fluoromount-G; SouthernBiotech).

### 2.6. Microscopy and Image Quantification

Immunocytochemical images were acquired using a Nikon Eclipse 80i fluorescence microscope and a DS-QiIMC charge-coupled device camera (Nikon). Cell counts were performed with NIS-Elements imaging software (Nikon) or ImageJ. PAX7 expression was calculated as the average percentage of PAX7-positive cells (%PAX7^+^) per total cells represented by Hoechst-positive cell nuclei. The number of MyHC^+^ cells was determined as the average percentage of Hoechst-positive nuclei surrounded by MyHC^+^ myotubes (%MyHC^+^) for each field of view. Myotube width was measured and presented as the average width of all myotubes per image field. To verify the reliability of our findings, we repeated 2-3 independent experiments for cell counting. For each staining, we prepared technical duplicates (2 coverslips), and at least 3 randomly selected fields per coverslip were photographed using a ×20 objective. The values of %PAX7^+^ and %MyHC^+^ were determined from at least 6 microscopic fields per experiment.

### 2.7. Sphere Growth Measurement and Calculation

EZ spheres were prepared from iPSCs and then dissociated and sorted as described above. Cells in the CD271^+^ fraction and CD15^–^ fraction were transferred into expansion medium to reform spheres. A parallelly prepared group of cells that did not go through MACS was used as control, referred to as “unsorted cells.” After 6 weekly passages by mechanical chopping, spheres were transferred into the cell freezing medium (C6295, Sigma-Aldrich) and stored in liquid nitrogen for 2 years. Cryopreserved spheres were then thawed and transferred into the expansion medium. After spheres were passaged once a week and maintained for 6 weeks, 8 spheres per group were transferred into the wells of a low-attachment 96-well plate on day 0. Each well contained one sphere with a similar size (~20 *μ*m diameter) in the expansion medium. On day 0, day 3, day 6, and day 9, diameters of the spheres were measured with the eyepiece reticle of the Nikon Eclipse TS 100 inverted microscope and used to calculate the sphere volume. Growth rates were calculated by normalizing each sphere's changes in volume relative to the initial volume on day 0. To verify the reliability of our findings, we repeated 3 independent experiments.

### 2.8. CD271 Gene Knockdown Using Small Interfering RNA (siRNA)

The improved culture expansion rate and myotube formation capacity in postsort CD271^+^ cells led to our specific interest in the potential involvement of CD271 expression in myogenesis. To answer this possibility, we examined whether silencing CD271 expression in human PSC-derived myogenic progenitors would cause any effects on myogenic differentiation. PSC-derived EZ spheres were dissociated, triturated in the terminal differentiation medium, and passed through a cell strainer as described above. CD271 small interfering RNA (siRNA) #1 and #2 or scrambled (5 *μ*M; J-009340-07, J-009340-10 and D-001810-10; Dharmacon, Lafayette, CO, USA) was transfected into 2.5 × 10^6^ cells in 100 *μ*l Human Stem Cell Nucleofector™ solution using the Amaxa Nucleofector™ 2b device (Lonza, Basel, Switzerland) with the program U-013. Transfected cells were plated onto coverslips and cultured in the terminal differentiation medium for 2 weeks. Cells were then fixed with ice-chilled methanol and immunolabeled for MyHC. A parallelly prepared group of cells was used to examine siRNA efficiency by western blotting at 6 hours after transfection. To verify the reliability of our findings, we conducted 2 independent experiments using EZ spheres derived from the iPSC line IMR-90 and ESC line WA-09 (H9).

### 2.9. Western Blot

Transfected cells were lysed in radioimmunoprecipitation assay buffer (RIPA buffer; EMD Millipore, Burlington, MA, USA) with a protease inhibitor cocktail (Thermo Fisher Scientific, Waltham, MA, USA) and 5 mM ethylenediaminetetraacetic acid (EDTA; Thermo Fisher Scientific). Protein concentrations were determined using the DC Protein Assay kit (Bio-Rad, Hercules, CA, USA). Proteins (20 *μ*g per lane) were run on a polyacrylamide gel and transferred onto a PVDF membrane (EMD Millipore). The membrane was immunoblotted with an anti-human CD271 antibody (345102, 1 : 100; BioLegend) followed by a secondary antibody conjugated with horseradish peroxidase (anti-mouse IgG HRP; Promega, Madison, WI, USA). Enhanced chemiluminescence substrate (Pierce Biotechnology, Waltham, MA, USA) was used to detect HRP on the immunoblot for chemiluminescence imaging using the UVP ChemStudio PLUS Imaging System (Analytik Jena USA, Upland, CA, USA).

### 2.10. Statistical Analysis

**Figure 3:**
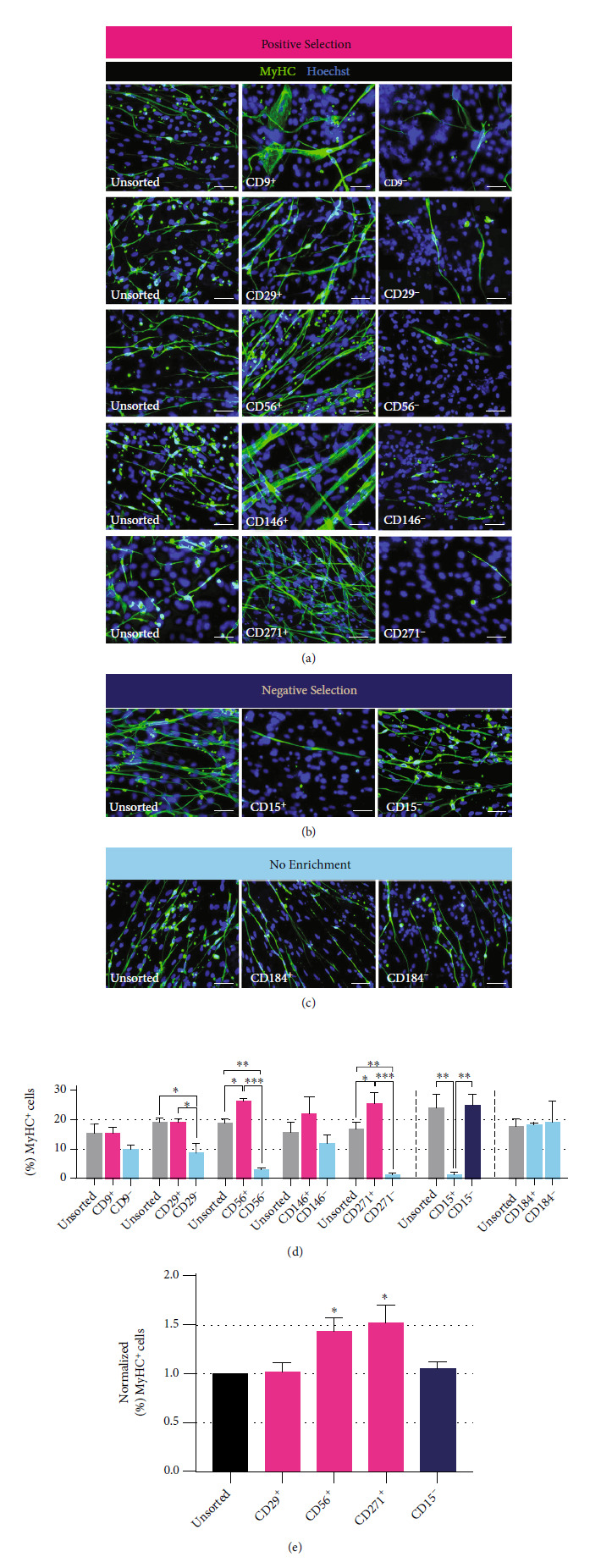
Myotube-forming efficiency of human iPSC-derived myogenic progenitors enriched using identified markers. (a–c) Representative ICC images of MyHC expression (stained green) in differentiated cultures of human iPSC-derived myogenic progenitors sorted using MACS. Scale bar = 50 *μ*m. (d) Means of % MyHC^+^ cells in differentiated cultures of unsorted, positive, and negative fractions. Error bars represent SEM from three independent experiments. Statistical significance was calculated by one-way ANOVA followed by Fisher's LSD post hoc test. ^∗^*p* < 0.05, ^∗∗^*p* < 0.01, and ^∗∗∗^*p* < 0.001. (e) Means of % MyHC^+^ cells of sorted fractions normalized by the % MyHC^+^ cells of parallel unsorted fractions. Error bars represent SEM from three independent experiments. Statistical significance was calculated by one-way ANOVA followed by Fisher's LSD post hoc test. ^∗^*p* < 0.05, significantly different compared to unsorted fractions.

**Figure 4:**
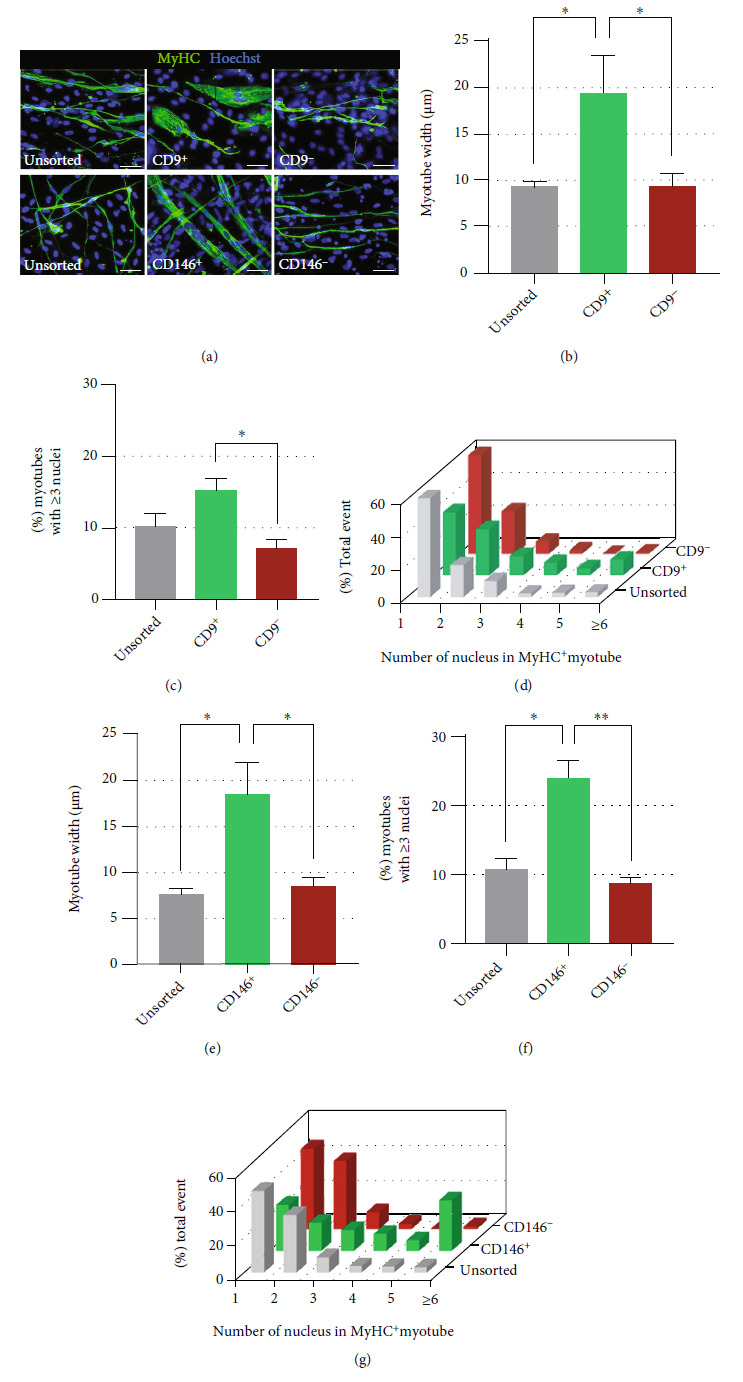
Characterization of myotubes formed by human iPSC-derived myogenic progenitors enriched using CD9 and CD146. (a) Representative ICC images of MyHC expression (stained green) in differentiated cultures of sorted human iPSC-derived myogenic progenitors. Scale bar = 50 *μ*m. (b–d) Properties of myotubes formed by differentiated cultures of unsorted cells and CD9^+^ and CD9^–^ human iPSC-derived myogenic progenitors: means of myotube width in microns, means of % myotube with ≥3 nuclei, and means of % myotube with 1 to ≥6 nuclei. (e–g) Properties of myotubes formed by differentiated cultures of unsorted and CD146^+^ and CD146^–^ human iPSC-derived myogenic progenitors: means of myotube width in microns, means of % myotube with ≥3 nuclei, and means of % myotube with 1 to ≥6 nuclei. Error bars represent SEM from three independent experiments. Statistical significance was calculated by one-way ANOVA followed by Fisher's LSD post hoc test. ^∗^*p* < 0.05, ^∗∗^*p* < 0.01.

**Figure 5:**
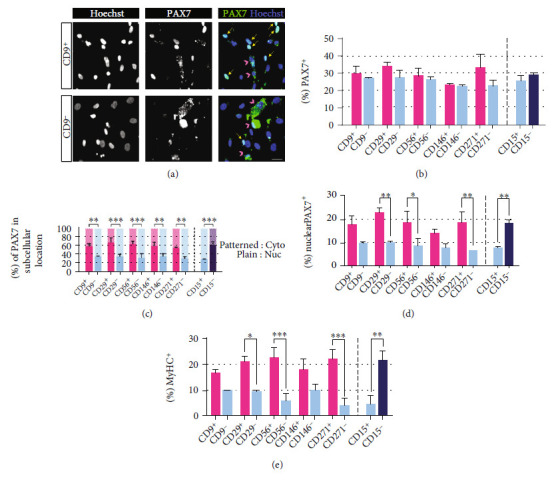
PAX expression level and distribution pattern of human iPSC-derived myogenic progenitors enriched using identified markers. (a) Representative ICC images of PAX7 expression (stained green) in undifferentiated cells of sorted human iPSC-derived EZ spheres. Long yellow arrows indicate nuclear localization of PAX7. Pink arrowheads indicate cytoplasmic distribution of PAX7. Scale bar = 20 *μ*m. (b) Means of % PAX7^+^ cells in undifferentiated cultures of sorted fractions. Error bars represent SEM from two independent experiments. No statistical significance as determined by one-way ANOVA. (c) Means of % cells with PAX7 expression localized in the nuclei and % cells with PAX7 expression scattered across the cytoplasm among PAX7^+^ cells in undifferentiated cultures of sorted fractions. Error bars represent SEM from two independent experiments. Statistical significance was calculated by one-way ANOVA followed by Bonferroni's post hoc test for pairwise comparisons. ^∗∗^*p* < 0.01, ^∗∗∗^*p* < 0.001. (d) Means of % nuclear PAX7^+^ cells in undifferentiated cultures of sorted fractions. Error bars represent SEM from two independent experiments. Statistical significance was calculated by one-way ANOVA followed by Bonferroni's post hoc test for pairwise comparisons. ^∗^*p* < 0.05, ^∗∗^*p* < 0.01. (e) Adapted from data in Figure 3(d), means of % MyHC^+^ cells in differentiated cultures of sorted fractions. Error bars represent SEM from three independent experiments. Statistical significance for pairwise comparisons was calculated by unpaired two-tailed Student's *t*-test. ^∗^*p* < 0.05, ^∗∗^*p* < 0.01, and ^∗∗∗^*p* < 0.001.

**Figure 6:**
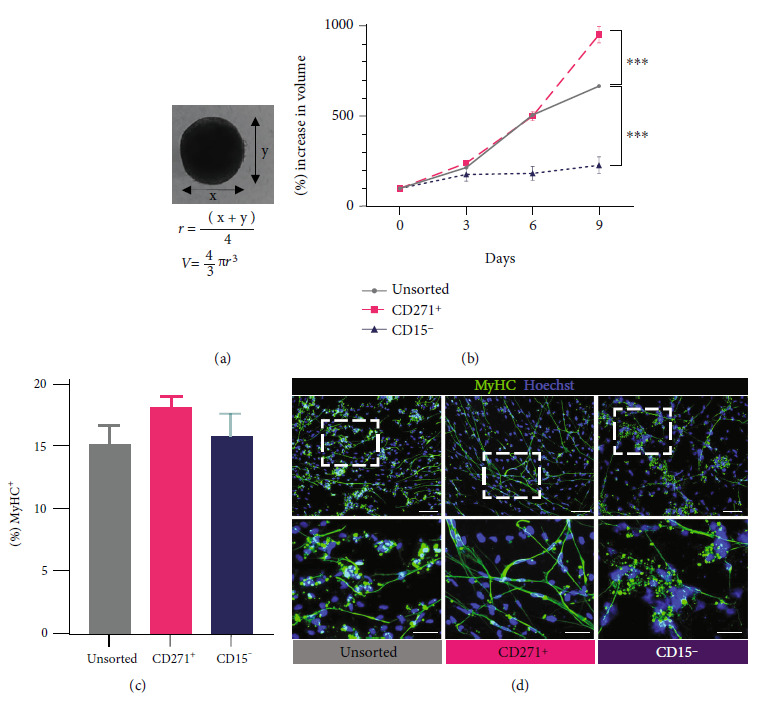
Purification of human iPSC-derived myogenic progenitors altered culture resilience to cryopreservation and expansion. (a) Measurements and formulas to calculate volume of each sphere. (b) Growth rate of sphere cultures prepared from cells in unsorted, CD271^+^, and CD15^–^ fractions after cryopreservation and culture expansion, presented as relative change in volume over 9 days in expansion medium. Error bars represent SEM from three independent experiments. Values on day 9 were analyzed by one-way ANOVA followed by Dunnett's post hoc test. ^∗∗∗^*p* < 0.001. (c) Means of % MyHC^+^ cells in differentiated cultures prepared from unsorted, CD271^+^, and CD15^–^ spheres after cryopreservation and expansion. Error bars represent SEM from three independent experiments. No statistical significance as calculated by one-way ANOVA. (d) Representative ICC images of MyHC expression (stained green) corresponding to (c). Top panel scale bar = 100 *μ*m; bottom panel scale bar = 50 *μ*m.

**Figure 7:**
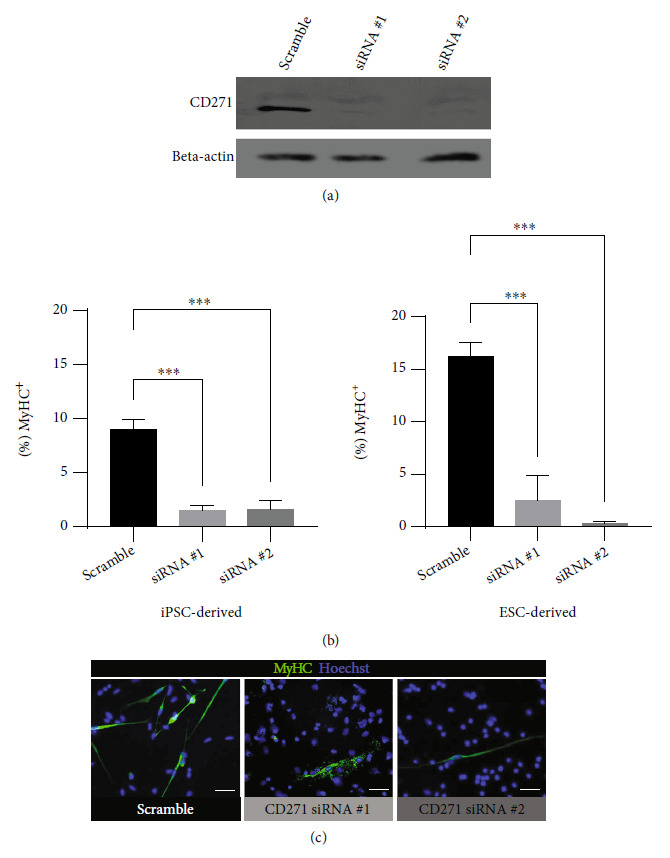
Knockdown of CD271 in human PSC-derived myogenic progenitors reduced and impaired myotube-forming ability. (a) Western blot analysis showed successful knockdown of CD271 levels after treatment with CD271 siRNA #1 and #2 compared to scrambled as a negative control. This antibody clone might recognize another posttranslational fragment, hence the faint bands in the CD271 blotting. Beta-actin served as the loading control. (b) Means of % MyHC^+^ cells in differentiated cultures prepared from cells treated with CD271 siRNA scrambled (negative control) #1 or #2. Error bars represent SEM from 6 analyzed images. Statistical significance was calculated by one-way ANOVA followed by Dunnett's post hoc test. ^∗∗∗^*p* < 0.001. (c) Representative immunocytochemistry images of MyHC expression (stained green) corresponding to (b). Scale bar = 20 *μ*m.

GraphPad Prism 9.1.0 (GraphPad Software, Inc., La Jolla, CA, USA) was used to perform statistical analyses. Quantitative data was graphed as means ± standard error of the mean (SEM) from at least two independent experiments. Unpaired two-tailed Student's *t*-test was performed to compare two groups. One-way ANOVA was performed to compare multiple groups. A statistically significant one-way ANOVA was followed up with the appropriate post hoc test: Fisher's least significant difference (LSD) test to compare each set of unsorted, positive, and negative fractions in Figures 3 and 4; the Bonferroni correction for pairwise comparison in Figure 5; and Dunnett's test to compare treated groups to control groups in Figures 6 and 7. Differences were considered significant when *p* < 0.05.

### 2.11. Flow Cytometry Analysis

EZ spheres were dissociated, passed through a 40 *μ*m filter, resuspended to a final concentration of 10^4^ cells per well in 96-well plates, incubated with antibodies (1 : 100) in FACS buffer (PBS(-) containing 1% BSA and 0.1% sodium azide) for 20 min on ice in the dark, and washed twice in FACS buffer. The following antibodies were used: BV421 anti-human CD15, PerCP/Cy5.5 anti-human CD184 (BioLegend), APC anti-human CD29; PE anti-human CD56, FITC anti-human CD146, and Alexa Fluor 647 anti-human CD271 (BD Biosciences). Stained cells were analyzed using BD LSRFortessa™ (BD Biosciences). Flow cytometry data were analyzed using FlowJo analysis software version 9.9.6 (BD Biosciences).

## 3. Results

### 3.1. Surface Marker Profiling of Human PSC-Derived Myogenic Progenitors by Antibody Screening

Human myogenic progenitors are derived from the human iPSC line IMR-90 and human ESC line WA09 (H9) using our sphere culture protocol. Traditional methods have used 10-20 ng/ml FGF-2 to drive the proliferation of cultured cells [30, 31], but FGF concentration required to maintain cells in pluripotency may vary from one cell line to another depending on culture conditions, ranging from as low as 4 ng/ml [32–35] to as high as 100 ng/ml [36–39]. We previously found that a high concentration (100 ng/ml) of FGF-2 significantly increased the number of human PSC-derived myogenic progenitors in EZ spheres, whereas the culture condition without FGF-2 produced barely any myogenic progenitors [6]. In this study, we compared the expression of surface markers between two preparations of EZ spheres derived from each line, cultured for 6 weeks in the expansion medium with or without 100 ng/ml FGF-2. EZ spheres were then dissociated into single cells, and 1.5 × 10^4^ cells per well were plated in 96-well glass round-bottom plates and cultured in the terminal differentiation medium overnight. Next, we used the BD Lyoplate™ Human Cell Surface Marker Screening Panel and stained the plated cells from FGF-2-treated EZ spheres with 255 antibodies against different surface markers. We expected to identify surface markers that were specifically expressed in human PSC-derived myogenic progenitors (Figure 1). FGF-2-treated cells were positive for 89 antibodies and negative for the other 166 antibodies (Figure 2(a) and Supplementary Figure S1A). Among the positive ones, CD29 and CD56 were obviously positive in the cells from FGF-2-treated EZ spheres (Figure 2(b)). This corresponds to the previous reports showing that these two markers are positive in various types of myogenic progenitors [2, 11–17, 19, 40, 41]. Therefore, we could use these two markers as positive staining to serve as a reference for immunostaining analysis. In contrast, immunocytochemistry results with antibodies against CD6 and CD83 are examples of negative staining (Figure 2(b)). CD6 is a type 1 transmembrane glycoprotein found on T lymphocytes that interacts with the ligand-activated leukocyte cell adhesion molecule (ALCAM) to activate T cell proliferation [42, 43], whereas CD83 is a member of the immunoglobulin superfamily that has primarily been reported as a marker of mature dendritic cells [44, 45].

We next compared the surface marker profiles between the cells derived from FGF-2-treated and nontreated (i.e., without FGF-2 supplementation) EZ spheres. Among the 89 antibodies stained positive in FGF-2-treated cells, 33 surface markers showed noticeable different expression levels compared to nontreated cells (Supplementary Figure S1B). While 10 showed higher expression in FGF-2-treated cells, 23 showed higher expression in nontreated cells (Figure 2(a) and Supplementary Figure S1C). For instance, the expression of CD271 was higher in FGF-2 treated cells, whereas CD15 expression was higher in non-FGF-2-treated control cells (Figure 2(c)). CD271 is a low-affinity neurotrophin receptor known to have critical functions in cell survival [46], differentiation [47], and migration [48] of neuronal cells. Next, we further narrowed down the markers of interest by confirming their expression on myogenic progenitors derived from multiple batches of human iPSC (IMR-90) and ESC (WA9/H9) lines. One of our selection criteria was that 30-70% of myogenic progenitor culture should stain positive across all batches tested. If the proportion of positive cells is higher or lower than the range between 30 and 70%, we considered that subsequent cell sorting would not make a significant change in the composition of the cell population. For example, CD9 fulfilled this criterion, whereas CD61 that stained positive in less than 30% cell population and CD49a that stained positive in more than 70% cell population did not. Taking into consideration the reproducibility and reliability of cell sorting, we selected markers that could be detected with high intensity and clear morphology when stained with immunocytochemistry. With a staining pattern that fulfilled our criterion, CD15 was a fitting candidate. On the contrary, CD95 did not fulfill our criterion (Figure 2(d)). Based on our initial selection, a series of markers (CD9, CD13, CD26, CD29, CD49b, CD49f, CD56, CD98, CD146, CD164, CD184, and CD271) were identified and used for further investigation in the next step.

### 3.2. Isolation of Human iPSC-Derived Myogenic Progenitors Using Identified Markers

To confirm specificity of the selected markers, we enriched myogenic progenitors from human iPSC-derived EZ spheres by MACS and then evaluated the potential of isolated cells for *in vitro* myotube differentiation. EZ spheres were prepared from iPSCs in the expansion medium supplemented with FGF-2. After the spheres were dissociated, single suspended cells were sorted by MACS using the selected surface markers. Sorted cells were plated down on coverslips and maintained in terminal differentiation medium (DMEM with 2% B27 supplement) for 14 days to induce myotube formation. As the efficiency of iPSC-derived myogenic progenitors often varied across batches, we used at least three independent preparations of EZ spheres to test the versatility of each marker. Immunostaining for MyHC indicated that positive (CD9, CD29, CD56, CD146, and CD271) and negative (CD15) markers could be used for myogenic progenitor enrichment (Figures 3(a) and 3(b)). On the contrary, CD184 did not work sufficiently for the isolation of myogenic progenitors as there is no substantial difference in myotube formation between positive and negative cell fractions following terminal differentiation (Figures 3(c) and 3(d)). Similarly, other markers, such as CD13, CD24, CD26, CD49b, CD49f, CD98, and CD164, also failed to enrich myogenic progenitors (Supplementary Figure S2). Additionally, we tested surface markers that were recently reported in previous publications but not listed in our screening panel, such as integrin *α*7 [2, 49], ErbB3 [19, 50], and CD82 [40, 51]. However, MACS using these markers could not enrich a specific population of myogenic progenitors from our EZ spheres (Supplementary Figure S2).

#### 3.2.1. Improved Myotube Formation Efficiency in CD29^+^, CD56^+^, CD271^+^, and CD15^–^ Fractions

When comparing the postsorting fractions of iPSC-derived FGF-2-treated cells, the number of MyHC^+^ myotubes was significantly higher in the differentiated cultures from CD29^+^, CD56^+^, CD271^+^, and CD15^–^ fractions (Figure 3(d)). As CD29 and CD56 are widely established as myogenic markers [11], it was important to evaluate the efficacy of these markers to enrich iPSC-derived myogenic progenitors prepared with our sphere-based culture protocol. Immunocytochemistry revealed that 19.4 ± 1.0% and 9.1 ± 3.4% cells were MyHC^+^ in the terminally differentiated cultures derived from the CD29^+^ fraction and CD29^–^ fraction, respectively (*p* < 0.05). Concurrently, the differentiated cells from the CD56^+^ fraction and CD56^–^ fraction had 26.6 ± 0.7% and 3.0 ± 0.7% MyHC^+^ cells, respectively (*p* < 0.001). The differentiated culture of the CD271^+^ fraction had more MyHC^+^ cells (25.6 ± 3.8%) compared to the CD271^–^ fraction (1.5 ± 0.4%, *p* < 0.001). Meanwhile, the CD15^+^ fraction and CD15^–^ fraction consisted of 1.3 ± 0.8% and 25.0 ± 3.8% MyHC^+^ cells following terminal differentiation, respectively (*p* < 0.01).

To determine whether cultures of CD29^+^, CD56^+^, CD271^+^, and CD15^–^ fractions showed higher myotube-forming capacity than their respective unsorted cell populations (control), we normalized the data for each individual experiment with their respective controls and calculated the average (Figure 3(e)). When comparing to the unsorted cell populations, only CD56^+^ and CD271^+^ fractions both displayed a significant increase in myotube formation (*p* < 0.05). These results indicate that cell sorting using CD56 and CD271 could enrich myogenic progenitor populations from iPSC-derived EZ spheres. On the flipside, although the ratio of MyHC^+^ cells is significantly higher in the CD29^+^ fraction compared to the CD29^–^ fraction, differentiation potential of cells in the CD29^+^ fraction and unsorted populations was not significantly different. Similarly, even though myogenic progenitors were found almost exclusively in the CD15^–^ fraction and barely any in the CD15^+^ fraction, differentiation potential of cells in the CD15^–^ fraction was not significantly different from cells in unsorted populations. As such, with the sorting technique we used, CD29 and CD15 did not sufficiently work for myogenic progenitor enrichment.

In addition, we were interested to determine how many CD marker-positive cells were originally distributed in the unsorted preparation of human PSC-derived EZ spheres (Supplementary Figure S3). For this analysis, we used both iPSC-derived and ESC-derived FGF-2-treated EZ spheres and compared the expression patterns of specific surface markers by FACS. We found that iPSC-derived and ESC-derived FGF-2-treated EZ sphere cells had similar expression patterns of CD29 and CD56. In contrast, while almost all iPSC-derived FGF-2-treated EZ sphere cells stained positive for CD271 with high intensity and negative for CD15, ESC-derived cells showed different expression patterns of CD271 and CD15: cells that stained positive for CD271 with two distinct intensities were detected, whereas a substantial proportion of cells stained positive for CD15 (Supplementary Figure S3).

#### 3.2.2. Increased Myotube Fusion and Myotube Width in CD9^+^ and CD146^+^ Fractions

As described above, myotube formation efficiency (% MyHC^+^ cells) was not significantly different between positive and negative fractions of EZ sphere cells following cell sorting based on the expression of CD9 and CD146. We proceeded to analyze the cellular morphology in their differentiated myotubes. Interestingly, we found that myotubes derived from CD9^+^ and CD146^+^ fractions were thicker and contained more nuclei per myotube compared to unsorted, CD9^–^, and CD146^–^ fractions (Figure 4(a)).

In the differentiated cultures from the CD9^+^ fraction, MyHC^+^ myotubes had an average width of 19.4 ± 4.0 *μ*m. These myotubes were significantly thicker when compared to the myotubes from the unsorted fraction (9.3 ± 0.5 *μ*m; *p* < 0.05) and CD9^–^ fraction (9.3 ± 1.3 *μ*m; *p* < 0.05) (Figure 4(b)). We next analyzed the fusion index in the differentiated myotubes, which characterized the number of cell nuclei per myotube. The proportion of MyHC^+^ myotubes containing ≥3 nuclei was significantly larger in the differentiated cultures of the CD9^+^ fraction (33.5 ± 3.9%) and CD9^–^ fraction (10.5 ± 2.7%; *p* < 0.05) (Figure 4(c)). The differentiated cultures of unsorted and CD9^–^ fractions barely yielded thickly fused myotubes with ≥6 nuclei (2.9 ± 2.9% and 0.9 ± 0.9%, respectively), whereas 9.7 ± 2.9% of total MyHC^+^ myotubes in the differentiated cultures of the CD9^+^ fraction contained ≥6 nuclei (Figure 4(d)).

Meanwhile, differentiated cultures of the CD146^+^ fraction yielded MyHC^+^ myotubes with an average width of 18.4 ± 3.6 *μ*m, significantly thicker than MyHC^+^ myotubes formed by differentiated cultures of the unsorted fraction (7.5 ± 0.7 *μ*m; *p* < 0.05) and CD146^–^ fraction (8.4 ± 1.1 *μ*m; *p* < 0.05) (Figure 4(e)). While CD9^+^ or CD146^+^ cells formed myotubes of similar widths, the myotubes derived from the CD146^+^ cell fraction were more highly fused and therefore contained more nuclei per myotube than the differentiated cultures of the CD9^+^ fraction. About half (57.32 ± 6.6%) of the total MyHC^+^ myotubes in the differentiated cultures of the CD146^+^ fraction contained ≥3 nuclei, exceeding the differentiated cultures of the unsorted fraction (19.5 ± 4.1%; *p* < 0.05) and CD146^–^ fraction (14.4 ± 2.2%; *p* < 0.01) by statistical significance (Figure 4(f)). Differences were even more apparent when comparing the proportion of total MyHC^+^ myotubes containing ≥6 nuclei, which was 3.2 ± 2.8%, 29.2 ± 4.1%, and 1.5 ± 0.8% in the differentiated cultures of unsorted, CD146^+^, and CD146^–^ fractions, respectively (Figure 4(g)).

#### 3.2.3. Increased Nuclear PAX7 Localization in CD9^+^, CD29^+^, CD56^+^, CD146^+^, CD271^+^, and CD15^–^ Fractions

Next, we determined whether the expression of an early myogenic progenitor marker PAX7 reflected the ability of myotube formation in each sorted fraction. For PAX7 analysis, we used two individual preparations of iPSC-derived EZ spheres. EZ spheres were dissociated, and single suspended cells were sorted by MACS based on the expression of the 6 selected surface markers (CD9, CD29, CD56, CD146, CD271, and CD15). Sorted cells were cultured overnight in a 24-well plate to allow for cell attachment onto coverslips and then fixed and immunolabeled for PAX7. Our detailed analysis of PAX7 immunostaining revealed that the sorted cells showed two distinct patterns of intracellular PAX7 distribution: localized to the nucleus (indicated by yellow arrows in Figure 5(a)) or widely scattered in the cytoplasmic region (white arrowheads in Figure 5(a)). There was a trend of increased total Pax7 expression in the fractions of CD9^+^, CD29^+^, CD56^+^, CD146^+^, CD271^+^, and CD15^–^ which contained higher proportions of total PAX7^+^ cells when compared to their respective counterparts, although the difference did not reach statistical significance (Figure 5(b)).

When we further analyzed subcellular localization in PAX7-positive cells, we found that PAX7 expression localized to the nuclei with significantly higher levels in all of the fractions where myogenic progenitors were enriched, whereas cells in their counterpart fractions contained more cytoplasmic PAX7 expression (Figure 5(c)). Specifically, nuclear PAX7 expression was identified in approximately 55-70% of PAX7^+^ cells of CD9^+^, CD29^+^, CD56^+^, CD146^+^, CD271^+^, and CD15^–^ fractions, but only in 29-35% of PAX7^+^ cells of CD9^–^, CD29^–^, CD56^–^, CD146^–^, CD271^–^, and CD15^+^ fractions. When disregarding cells with cytoplasmic PAX7 expression and only comparing the overall proportions of nuclear PAX7^+^ cells, we noticed that the undifferentiated cultures of CD29^+^, CD56^+^, CD271^+^, and CD15^–^ fractions contained significantly higher ratios of nuclear PAX7^+^ cells than their counterparts CD29^–^, CD56^–^, CD271^–^, or CD15^+^ fractions (Figure 5(d)). When we regraphed the percentages of MyHC^+^ cells in differentiated cultures of these 12 sorted fractions from earlier experiments in Section 2.1 (Figure 5(e)), these results had a similar overall pattern compared to the percentages of nuclear PAX7^+^ cells in undifferentiated cultures of these 12 sorted fractions. However, nuclear PAX7 expression in undifferentiated cultures of sorted fractions is not perfectly predictive of myotube-forming ability of differentiated cultures of sorted fractions, as nuclear PAX7 expression levels in undifferentiated cultures of sorted fractions did not faithfully mirror MyHC expression levels in differentiated cultures of sorted fractions.

#### 3.2.4. The Effects of Cryopreservation and Culture Expansion on Postsort Cells from CD271^+^ and CD15^–^ Fractions

We experienced that cryopreservation and long-term culture often influence the proliferation of human PSC-derived myogenic progenitors in EZ spheres. Therefore, we determined whether depleting CD271^–^ or CD15^+^ cells from our cultures would affect their resilience to cryopreservation and frequent expansion over a prolonged duration. After expanding iPSC-derived EZ spheres up to 16 weekly passages, we dissociated the spheres and sorted the cells by MACS based on CD271 or CD15 expression. Cells from the CD271^+^ fraction and CD15^–^ fraction were transferred back into the expansion medium to reform sphere cultures. We referred to these groups of cells as “CD271^+^ spheres” and “CD15^–^ spheres,” while a parallelly prepared group of cells that did not go through sorting will be referred to as “unsorted spheres.” These three groups of spheres were expanded through 6 weekly passages, cryopreserved, and then thawed and expanded through another 6 weekly passages. At this point, these spheres had been passaged 30 times. Individual spheres of similar sizes were isolated and cultured in the 96-well plate. Perpendicular diameters of each sphere were measured at days 0, 3, 6, and 9 to calculate the volume (Figure 6(a)). Increase in sphere size indicates higher proliferation rate than cell death rate. The growth rate of undifferentiated spheres in each group was graphed (Figure 6(b)) [52]. From day 0 until day 6, the growth rate of CD271^+^ spheres mirrored that of unsorted spheres. At day 9, the growth rate of CD271^+^ spheres had surpassed that of unsorted spheres (997 ± 146% and 728 ± 86% of their initial sizes, respectively; *p* < 0.001). Unexpectedly, CD15^–^ spheres had a relatively stunted growth rate that became apparent at day 6 compared to the other two groups, and the average size at day 9 was only 309 ± 45% of their initial size, which was a dramatic difference compared to unsorted spheres (*p* < 0.001).

We next examined whether these three groups of spheres would differ in myotube-forming abilities when differentiated. Cells from these spheres were plated down on coverslips and terminally differentiated for two weeks. Differentiated cultures of CD271^+^ spheres and CD15^–^ spheres displayed similar ratios of myotube-forming cells compared to unsorted spheres (18.14 ± 1.67%, 15.83 ± 1.81%, and 15.17 ± 2.42%, respectively) (Figure 6(c)). Morphologically, differentiated cultures of CD271^+^ spheres appeared the healthiest with long, aligned myotubes and barely any clustered aggregates of cells. Differentiated cultures of both unsorted spheres and CD15^–^ spheres also contained healthy-looking myotubes, although visibly shorter and less aligned than that of CD271^+^ spheres. Clustered aggregates of cells were present in differentiated cultures of both unsorted spheres and CD15^–^ spheres; cells in the latter group formed noticeably larger clusters (Figure 6(d)).

#### 3.2.5. The Importance of CD271 Expression in Terminal Differentiation of Human PSC-Derived Myogenic Progenitors

To investigate whether CD271 expression plays critical roles for myogenic differentiation, we silenced CD271 in human PSC-derived myogenic progenitors using RNA interference. EZ spheres derived from human iPSC (IMR-90) and ESC (WA9/H9) lines were dissociated into single suspended cells and transfected with two different sequences of CD271 siRNA (#1 and #2) or scrambled. At 6 hours after transfection, cells were lysed and analyzed for CD271 protein levels. Western blot results showed successful knockdown of CD271 levels by siRNA #1 and #2, whereas scrambled served as a negative control (Figure 7(a)). Parallelly prepared cells were plated down on coverslips at 24 hours after transfection and cultured in the terminal differentiation medium for two weeks. MyHC immunostaining showed that myotube formation was significantly reduced in the differentiated cells which CD271 mRNA had been knocked down when compared to the cells transfected with scrambled siRNA (Figure 7(b)). Of note, myotubes formed by cells treated with siRNA #1 displayed apparent morphological defects (Figure 7(c)). Together, the reduction and impairment of myotube formation following CD271 knockdown may imply essential roles of CD271 expression for skeletal muscle development and differentiation.

## 4. Discussion

Strict maneuver of the cell differentiation process and isolation of desired cell types are fundamental to the successful use of human PSC derivatives for *in vitro* modeling or cell-based therapy. Not only that human biopsy-derived and PSC-derived myogenic progenitors do not exhibit fully equivalent properties, but significant phenotypic discrepancies were also observed even within human PSC-derived myogenic progenitors prepared via different methods. Sorted cells may also undergo changes in their surface marker profiles during expansion and differentiation in culture. This may explain the limited success and the lack of consensus on the enrichment of human PSC-derived myogenic progenitors. Even so, recent advances enabling the isolation of human myogenic progenitors based on surface marker expression have facilitated the analyses of their cell identities and myogenic potential.

We previously reported that myogenic progenitor populations derived from human PSCs using our transgene-free, serum-free protocol do not all homogenously express myogenic factors PAX7, MyoD and Myogenin [6, 9]. In this study, we described distinct cell types within the human PSC-derived EZ sphere cell populations. We performed the screening of surface marker expressions using myogenic progenitors derived from both human iPSC and ESC lines. After the surface marker profiles were established, we performed further investigation using iPSC-derived myogenic progenitors because accurate disease modeling using patient-specific cell lines demands efficient purification of iPSC-derived myogenic progenitors. Although MACS does not generate graphical representation of cell-specific multiple-marker profiles that FACS could provide through a flow chart, we employed MACS to isolate the cells due to its several pros over FACS that better fulfill our demands for the single-marker sorting in this study: MACS is simpler, 4-6 times quicker, can process larger number of cells at once due to its ability to run samples in parallel, and produces higher cell survival with only 7-9% cell loss compared to ~70% cell loss for FACS [21, 53] as well as higher postsort population growth [22]. Purity of postsort cells yielded by MACS has been reported to be in the range of 80-90% [21, 22, 54–56], and can be improved by using more antibodies and magnetic microbeads than recommended by manufacturer protocols [21]. Multiple-marker sorting can also be performed sequentially with MACS. Enrichment of muscle progenitors using the 6 surface markers identified in this study enabled differentiation and maturation of human iPSC-derived FGF-2-treated EZ sphere cells with significantly greater *in vitro* myogenic potential. Protein names and representative biological roles of these 6 markers are summarized in Table 1.

As predicted, well-established human myogenic markers CD56 (neural cell adhesion molecule; NCAM) and CD29 (integrin beta-1) were able to isolate most myogenic progenitors from our EZ sphere cultures. Several groups have reported successful enrichment of human PSC-derived myogenic cells with CD56 [19, 40] and CD29 [57]. The ratio of myotube-forming cells in CD56^+^ fraction was higher than not only CD56^–^ fraction but also unsorted populations with great significance, implying that CD56 alone may sufficiently work for enrichment of human PSC-derived myogenic progenitors. CD29 on the other hand is insufficient as a sole marker to identify human myogenic progenitors, as CD29^–^ fraction still contained a substantial proportion of myotube-forming cells. This finding is consistent with the previously suggested notion that not all myogenic progenitors express CD29 [11, 12]. Nonetheless, higher myogenic potential within CD29^+^ cell populations implies that CD29 expression may have a role in promoting muscle differentiation. In our study, myogenic potential was not significantly different between CD29^+^ and unsorted populations following differentiation. Eliminating CD29^–^ cells might not change the cell population makeup much, since most of the unsorted cells were CD29-postive (Figure S3).

Also known as p75 neurotrophin receptor or low-affinity nerve growth factor receptor (p75NGFR), CD271 is a very promising marker to isolate human PSC-derived myogenic progenitors and holds tremendous potential to unveil novel specifics about the mechanisms of myogenesis. Previously, mixed results were reported for the enrichment of PSC-derived myogenic progenitors using CD271 as a positive marker: one group reported unsuccessful CD271-based enrichment of myogenic progenitors from PAX7/Myf5 dual reporter ESCs cultured in a serum-containing medium [20], while another two groups successfully used CD271-based enrichment of iPSC-derived myogenic progenitors prepared under serum-free culture conditions [19, 50]. In the present study, CD271-based enrichment of myogenic cells was successful in human iPSC-derived EZ spheres prepared under serum-free protocol: myotube-forming cells were found almost exclusively in the CD271^+^ fraction; negligible ratio of myotube-forming cells in the CD271^–^ fraction probably reflects contamination with the CD271^+^ cells. Although it is not easy to decode why there were various results on driving myogenic commitment following CD271-based enrichment, the differences of derivation protocols and PSC lines between studies may explain diverse responses to each protocol. It is intriguing that since almost all cells within unsorted populations in iPSC-derived EZ spheres stained positive for CD271 with high intensity, theoretically cellular composition was not largely different between the unsorted populations and CD271^+^ fraction populations, yet CD271^+^ fraction exhibited a significantly higher ratio of myogenic differentiation compared to the unsorted populations. We speculate that CD271^–^ cells might have inhibitory effects on the process of muscle differentiation in CD271^+^ cells. This hypothesis may also be supported by our finding that postsorted CD271^+^ spheres exhibited higher growth rate than unsorted spheres after cryopreservation and long-term culture expansion. These CD271^+^ sphere cells also retained status as myogenic progenitors with a higher ability of myotube formation following terminal differentiation when compared to the unsorted populations. Meanwhile, silencing CD271 expression resulted in reduced myotube formation and defective myotube morphology, confirming that CD271 is requisite for human muscle differentiation.

The function of CD271 in rodent myoblasts has been elucidated in several reports. When supplemented with neurotrophins which support the survival and differentiation of neurons, CD271^+^ primary and C2C12 mouse myoblasts proliferated but did not differentiate, while showing increased levels of phosphorylated inhibitor of nuclear factor kappa B (I-*κ*B) and protein kinase B, both of which promote cell survival [58]. CD271 was also reported to inhibit the differentiation of C3H10T1/2 mouse multipotent mesenchymal stem cells into osteoblasts, adipocytes, chondrocytes, and myocytes [59]. In L6C5 rat myogenic cells, CD271 was shown to promote myotube differentiation and fusion through the involvement of nuclear factor kappa B (NF-*κ*B) activation and the small heat shock proteins (sHSPs) *α*B-crystallin and Hsp27 [60]. Another study reported that CD271 expression in L6C31 rat myoblasts was detected early after plating in growth medium but started diminishing after switching to differentiation medium and was completely undetected in myotubes. This same study reported that in primary cultures of adult human myoblasts, some CD271^+^ cells were present at all timepoints tested, from early after plating in growth medium until after 6 days in differentiation medium [61]. Changes in CD271 expression have been reported during transitions from early to late waves of human fetal myogenesis, which also correlates to the development of primary limb myofibers or maturation of secondary fetal myofibers [19]. CD271/ErbB3-based isolation can be used to enrich Pax7^+^ cells and Myf5^+^ cells from human fetal muscles during the first and second trimesters of gestation, including at time points before any other myogenic surface markers are presented on muscle progenitors [51]. A CD271^+^/ErbB3^+^ subpopulation emerged in the muscle with expression of myogenic transcription factors at 8 weeks of gestation; this subpopulation began to co-express some cell surface markers such as CD56, CD82, and CD146 at 11 weeks of gestation, then started losing CD271 expression at 16 weeks of gestation [19]. CD271 may also play a crucial role in muscle fiber type determination, as a study utilizing mouse myoblasts showed that CD271 facilitates the induction of fast myosin heavy chain and of fast-glycolytic markers [62]. Impaired oxidative metabolism and a concurrent buildup of CD271 proteins in mdx mice observed in this study parallels the increased levels of nerve growth factor transcripts in patients of Duchenne Muscular Dystrophy, thus the authors suggested that hyperactivation of the CD271-associated pathway may rescue the slow-oxidative phenotype in the dystrophic pathology. Taken together, the data indicate that CD271 expression in skeletal muscle mediates the survival and proliferation of myoblasts as well as their differentiation and fusion into myotubes. How the mechanisms of CD271-associated pathway are similarly or differently regulated in myogenesis of rodents and human remains to be determined.

A recent study revealed that ESC-derived CD271^high^ myogenic progenitors expressed significantly higher levels of myogenic factors PAX7, MYF5, MYOD1 and MYOG, and displayed higher myotube-forming ability compared to CD271^low^ cells [41]. FGF-2-treated ESC-derived EZ sphere cells in our study seemed to exhibit similar distinction of CD271^high^ and C271^low^ subpopulations on FACS analysis, where we could see two peaks representing a subset of cells that stained positive for CD271 with high intensity and another subset with lower intensity (Supplementary Figure S3). Detection of CD271^high^ and CD271^low^ cells has also been reported in various resident human cell types, such as hypopharyngeal cancer cells, brain melanoma cells, breast epithelial progenitors, medulloblastoma cells and pancreatic stellate cells [63–69]. CD271^high^ cells in these studies are cancerous and highly proliferative. Further characterization of the differences between CD271^high^ and CD271^low^ populations in various cell types would allow better understanding on possible roles of CD271 in pluripotency and lineage commitment.

Our current data supports the idea that CD15 could serve as a negative marker for myogenic progenitors derived from human pluripotent stem cells. We found that CD15^+^ cells barely possessed any myogenic activity, whereas CD15^−^ fraction yielded a highly enriched population of robustly myogenic progenitors. However, CD15^−^ selection did not result in a muscle differentiation level higher than unsorted populations. Similar to the arguments above for CD29-based selection, this is probably because the proportion of CD15^+^ cells was very low in EZ spheres and CD15^+^ cell removal did not influence the entire composition. Furthermore, the presence of CD15^+^ cells might not have such inhibitory impact on myogenic differentiation of the remaining cells in the populations, which is unlike what we proposed above about CD271^−^ cells. Nonmyogenic CD15^+^ cells might even have supporting roles for the maintenance of CD15^−^ myogenic cells, as postsorted CD15^−^ spheres expanded at a significantly diminished rate after cryopreservation and long-term culture expansion compared to parallelly prepared unsorted cells. Noticeably larger clustered aggregates of cells were found in differentiated cultures of CD15^–^ sphere cells, although these CD15^−^ cells retained status as myogenic progenitors with myotube-forming ability comparable to the unsorted cells. Further studies are required to elucidate what type of cells express CD15 in our EZ sphere cultures. Several groups reported that CD15^+^ cells are predominantly detectable in the adipogenic populations with relatively less myogenic capacity, whereas highly myogenic progenitor cells lack CD15 expression [13, 14, 70].

We here found CD9 and CD146 as new surface proteins, which may be pivotal to human myotube maturation and fusion. CD9 and CD146 are unbefitting for the isolation of myogenic progenitors derived using our protocol, as myotube-forming cells were similarly distributed in CD9^+^ and CD9^−^ fractions as well as in CD146^+^ and CD146^−^ fractions. However, myotubes formed by CD9^+^ cells and CD146^+^ cells were thicker and more multinucleated than myotubes formed by CD9^−^ cells and CD146^−^ cells. More specifically, some myotubes from the CD9^+^ fraction had an unusually high width to length ratio, with nuclei clustered in the center instead of lining up the major axis of the myotubes (Figures 3 and 4). Peculiarly, similar morphology has been observed in mouse cells lacking CD9. In culture, myoblasts from CD9^−/−^ mice fused with a higher frequency than normal myotubes to form large syncytia that contained numerous nuclei in culture. Additionally, CD9^−/−^ mice showed abnormal muscle regeneration featured by the formation of discrete giant dystrophic myofibers [71]. An earlier study reported that anti-CD9 monoclonal antibodies inhibited and delayed differentiation of mouse myoblast C2C12 cells into elongated myotubes [72]. Despite these discrepancies that could be explained by biochemical incompatibilities of different species, the comparable observations of abnormal myotube fusion indisputably link tetraspanin CD9 with myogenesis. In fact, it has been implicated that upregulation of CD9 expression activates mobilization of murine stem cells to the injured muscles [73]. Although a recent study defined Lineage^−^/*α*7integrin^+^/CD9^+^ cells as the myogenic compartment within murine adult muscle stem cells and myoblasts [74], CD9 has never been proposed as a human myogenic progenitor marker. The regulatory effects of CD9 on different adhesion molecules in the immune system [75] and in platelet recruitment [76] suggest its importance in cellular activities triggered by injury response. We speculate that CD9 may play a similar role in the migration, adhesion and aggregation of myogenic cells at injury sites. More work is warranted to pinpoint the possible involvement of CD9 in myotube fusion in humans. Not only in CD9-sorted cells, CD146^+^ myotubes were also thicker and had rows of centrally located nuclei, while highly aligned and fused with adjacent myotubes. CD146^+^ cells isolated from adult muscle, fetal muscle and placenta were previously reported to exhibit high myogenic potential [11]. *In situ*, CD146^+^ cells were found in the endomysium in adult muscle [13], as well as adjacent to myofibers and within blood vessels in fetal skeletal muscle [77]. Considering the resident location of CD146^+^ cells and their myogenic potential, it is probable that CD146 marks a subset of cells responsible for migration, organization, and alignment of myotube-forming cells.

PAX7 as a transcription factor is mainly found in the nuclei of myogenic progenitors, but our detailed analyses revealed that PAX7 expression was also detected within the cytoplasmic compartment. Specific functions and posttranslational events of transcription factors can be inferred through their subcellular localization, but there is a lack of studies on PAX7 regulation in myogenic progenitors in terms of protein distribution and transporting. One study reported that dfd13 dystrophin-deficient mouse myoblasts had a significantly higher percentage of PAX7 which was localized in the cytoplasm compared to C2C12 wild-type mouse myoblasts [78]. The detailed analysis of subcellular distribution revealed that while cytoplasmic PAX7 was localized in the endoplasmic reticulum, Golgi, and recycling endosome in both dfd13 and C2C12 myoblasts, PAX7 localized in recycling endosomes was significantly higher in dfd13 myoblasts, indicating that PAX7 was more actively transported across the cytoplasm in dystrophin-deficient myoblasts. This study identified that PAX7 interacts with KPNA2 (karyopherin-*α*2, or importin subunit alpha-1), a member of the family of proteins which function as protein transporters to the nucleus, confirming the preposition by an earlier study that KPNA2 is important for myoblast proliferation [79]. Another myogenic factor synthesized in the cytoplasm and rapidly transported to the nucleus is MyoD; degradation of MyoD occurs both in the nucleus and in the cytoplasm [80]. As such, distribution pattern and translocation of Pax7 may be crucial to determine whether this protein has been properly activated to function and properly degraded afterwards. Scattered cytoplasmic distribution of PAX7 observed in our study may characterize different cell subtypes with different PAX7 events and functions, or there could be a defect in PAX7 protein trafficking in or out of the nucleus. Expectedly, sorted fractions of undifferentiated iPSC-derived EZ sphere cells with higher nuclear PAX7 localization correspond to fractions that when differentiated produced more myotubes (CD29^+^, CD56^+^, CD271^+^, CD15^–^) or higher myotube fusion (CD9^+^, CD146^+^). Note that CD56^+^ fraction and CD271^+^ fraction contained more nuclear PAX7^+^ cells than their counterparts CD56^–^ fraction and CD271^–^ fraction with only moderate significance (*p* < 0.05 and *p* < 0.01, respectively), but the former group produced more myotubes than the latter group with high significance (*p* < 0.001). The difference in statistical difference between these two pairs of sorted fractions may indicate an increase in muscle differentiation commitment or ability after depleting CD56^–^ cells and CD271^–^ cells from the cultures. This may explain why CD56^+^ fraction and CD271^+^ fraction were the only preparations that formed significantly more myotubes than their unsorted controls (Section 2.1). Successful identification of surface markers that can isolate human PSC-derived myogenic progenitors with higher potential for myotube formation and fusion proves our approach feasible [11]. An enrichment using these identified surface markers allows us to characterize different cell subtypes within myogenic progenitor pools and how they interact with each other, which in turn allows the investigation of how the expression of these surface markers regulates myogenesis. Using surface markers for enrichment, we can delay replicative senescence, increase proliferation, and improve muscle differentiation of myogenic progenitors, thereby facilitating more convenient, productive, and efficient utilization of these cells in research. We mainly focused on *in vitro* evaluations in the current study, so we have not yet examined the potential of enriched cells for *in vivo* muscle regeneration following xenotransplantation. Our future research directions include assessing the engraftment capacity of enriched human PSC-derived myogenic progenitors to determine whether there are prospects for use of these identified markers in transplantation studies. In terms of *in vitro* characterization, the need to mature progenitors even more for functional and mechanistic analysis remains a challenge to be addressed in the field of muscle biology research.

## 5. Conclusion

Cell surface marker profiling has proven useful for the enrichment of myogenic progenitors derived from human PSCs via transgene-free method, as well as the identification of previously unreported surface proteins that may have an essential role in myotube formation and maturation. Better characterization of the cellular traits of human myogenic progenitors prepared via a directed differentiation method could lead to *in vitro* models that recapitulate human development and cell-specific lineages more closely, thus allowing for enhanced clinical utility. Furthering our knowledge of phenotypically distinct cells that possess myogenic progenitor properties and function could also conceivably aid the selection of promising targets to provide inroads to regenerative approaches for muscular diseases and muscle injuries. We anticipate that more studies will be done to define and distinguish the subpopulations of human PSC-derived myogenic progenitors, thereby facilitating the discovery of novel insights into human muscle development, homeostasis, aging, and disease.

## Figures and Tables

**Table 1:** Selected markers and their known biological involvements in human cells.

Markers	Aliases	Known biological involvements in human cells
CD9	(i) Tetraspanin-29 (TSPAN29) (ii) Leukocyte antigen MIC3 (iii) Mobility-related protein-1 (MRP1)	(i) Sperm-egg fusion [81, 82] (ii) Migration of immune cells [83] (iii) Platelet activation and aggregation [84]
CD29	(i) Integrin beta-1 (ITGB1) (ii) Fibronectin receptor subunit beta (FNRB) (iii) Very late antigen beta (VLAB)	(i) Proliferation, apoptosis, and migration of trophoblasts [85] (ii) Angiogenesis and lymphangiogenesis [86] (iii) Adhesion of osteoblasts to collagen, laminin, and fibronectin [87] (iv) Myogenesis [11, 88]
CD56	(i) Neural cell adhesion molecule (NCAM)	(i) Immune system development and function [89] (ii) Neurogenesis and neurite outgrowth [90] (iii) Myogenesis [11, 91]
CD146	(i) Melanoma cell adhesion molecule (MCAM or MelCAM) (ii) MUC18	(i) Angiogenesis [92] (ii) Migration and bone formation of mesenchymal stem cells [93] (iii) Fetal [94] and postnatal [95] myogenesis
CD271	(i) p75 neurotrophin receptor (p75^NTR^) (ii) Low-affinity nerve growth factor receptor (LNGFR)	(i) Differentiation [47] and migration [48] of neural cells (ii) Odontogenic differentiation of mesenchymal stem cells [96] (iii) Fetal myogenesis [50]
CD15	(i) Stage-specific embryonic antigen 1 (SSEA-1) (ii) Sialyl Lewis^X^ (sLeX)	(i) Sperm-egg fusion [97] (ii) Adhesion of leukocytes to endothelial cells and platelets [98] (iii) Adipogenesis [11]

## Data Availability

The data used to support the findings of this study are included within the article. We freely accept requests for access to these data, which should be made to the correspondence author (Masatoshi Suzuki, masatoshi.suzuki@wisc.edu).

## Additional Points

*Summary Statement.* Cell surface markers CD9, CD15, CD29, CD56, CD146, and CD271 can be used to enrich human skeletal muscle progenitor cells derived from pluripotent stem cells.

## Supplementary Materials

Supplementary Materials Supplementary Table S1: antibodies used in this study. Supplementary Figure S1: surface marker profile of human iPSC-derived myogenic progenitors. (A) 89 surface markers that were not detected in FGF-2-treated cells. (B) 56 surface markers that did not show noticeable different expression levels between FGF-2-treated and nontreated cells. (C) 10 surface markers that showed higher expression in FGF-2-treated cells and 23 that showed lower expression in FGF-2-treated cells. Supplementary Figure S2: surface markers that failed to enrich human iPSC-derived myogenic progenitors. A visibly similar number of myotubes were found in both positive and negative fractions of human iPSC-derived EZ sphere cells sorted based on their expression of CD13, CD24, CD26, CD49b, CD49f, CD82, CD98, CD164, ErbB3, and integrin *α*7; these markers were unable to enrich myogenic progenitors. Scale bar = 20 *μ*m. Supplementary Figure S3: surface marker profiling of human PSC-derived myogenic progenitors by flow cytometry. Expression of positive markers CD29, CD56, CD146, and CD271, negative marker CD15, and “no enrichment” marker CD184 (from left to right) in human iPSC-derived (top) and ESC-derived (bottom) FGF-2-treated EZ sphere cells.
