# Supplementary material for: Cell Surface Proteins for Enrichment and In Vitro Characterization of Human Pluripotent Stem Cell-Derived Myogenic Progenitors
Source: Stem Cells Int. 2022 Feb 24;2022:2735414. doi: 10.1155/2022/2735414 (PMC8894063; doi:10.1155/2022/2735414)
Supplement: Supplementary Materials — Supplementary Table S1: antibodies used in this study. Supplementary Figure S1: surface marker profile of human iPSC-derived myogenic progenitors. (A) 89 surface markers that were not detected in FGF-2-treated cells. (B) 56 surface markers that did not show noticeable different expression levels between FGF-2-treated and nontreated cells. (C) 10 surface markers that showed higher expression in FGF-2-treated cells and 23 that showed lower expression in FGF-2-treated cells. Supplementary Figure S2: surface markers that failed to enrich human iPSC-derived myogenic progenitors. A visibly similar number of myotubes were found in both positive and negative fractions of human iPSC-derived EZ sphere cells sorted based on their expression of CD13, CD24, CD26, CD49b, CD49f, CD82, CD98, CD164, ErbB3, and integrin α7; these markers were unable to enrich myogenic progenitors. Scale bar = 20 μm. Supplementary Figure S3: surface marker profiling of human PSC-derived myogenic progenitors by flow cytometry. Expression of positive markers CD29, CD56, CD146, and CD271, negative marker CD15, and “no enrichment” marker CD184 (from left to right) in human iPSC-derived (top) and ESC-derived (bottom) FGF-2-treated EZ sphere cells. [file 2735414.f1.docx]

**Supplementary Table S1.** Antibodies used in this study.

| Antibody | Host | Clone | Catalog # | Vendor |
| --- | --- | --- | --- | --- |
| BD Lyoplate™ Human Cell Surface Marker Screening Panel | Mouse, rat |  | 560747 | BD Biosciences |
| CD9-FITC | Mouse | M-L13 | 555371 | BD Biosciences |
| CD15-FITC | Mouse | HI98 | 555401 | BD Biosciences |
| CD26-FITC | Mouse | M-A261 | 555436 | BD Biosciences |
| CD29-APC | Mouse | MAR4 | 561794 | BD Biosciences |
| CD29-BB515 | Mouse | MAR4 | 564565 | BD Biosciences |
| CD49b-FITC | Mouse | AK7 | 555498 | BD Biosciences |
| CD49f-FITC | Rat | GoH3 | 130-097-245 | BD Biosciences |
| CD55 | Mouse | IA10 | 555691 | BD Biosciences |
| CD56-FITC | Mouse | B159 | 562794 | BD Biosciences |
| CD56-PE | Mouse | MY31 | 556647 | BD Biosciences |
| CD98-FITC | Mouse | UM7F8 | 556076 | BD Biosciences |
| CD146-FITC | Mouse | P1H12 | 560846 | BD Biosciences |
| CD164-FITC | Mouse | N6B6 | 551297 | BD Biosciences |
| CD271-AlexaFluor647 | Mouse | C40-1457 | 560326 | BD Biosciences |
| CD271-BB515 | Mouse | C40-1457 | 564580 | BD Biosciences |
| EGFR | Mouse | EGFR.1 | 555996 | BD Biosciences |
| CD15-BV421 | Mouse | W6D3 | 740086 | Biolegend |
| CD184-PerCP/Cy5.5 | Mouse | 12G5 | 306515 | Biolegend |
| ITGN-α7-FITC | Rabbit |  | orb14076 | Biorbyt |
| CD34 | Mouse | 4H11 | 11-0349-41 | Invitrogen |
| CD331 | Rabbit | D8E4 | 9740S | Cell Signaling Technology |
| CD332 | Rabbit | D4H9 | 11835S | Cell Signaling Technology |
| CD333 | Rabbit | C51F2 | 4574S | Cell Signaling Technology |
| CD334 | Rabbit | D3B12 | 8562S | Cell Signaling Technology |
| CD15-Microbeads | Mouse | VIMC6 | 130-046-601 | Miltenyi Biotec |
| CD56-Microbeads | Mouse | AF12-7H3 | 130-050-401 | Miltenyi Biotec |
| CD13-FITC | Cell line | REA263 | 130-103-732 | Miltenyi Biotec |
| CD24-FITC | Mouse | 32D12 | 130-099-118 | Miltenyi Biotec |
| CD29-FITC | Mouse | TS2/16 | 130-101-255 | Miltenyi Biotec |
| CD82-FITC | Cell line | REA221 | 130-101-309 | Miltenyi Biotec |
| CD184-APC | Mouse | 12G5 | 130-100-070 | Miltenyi Biotec |
| CD271-PE | Mouse | ME20.4-1.H4 | 130-099-023 | Miltenyi Biotec |
| ErbB3-Vio Bright FITC | Cell line | REA508 | 130-107-965 | Miltenyi Biotec |
| Anti-FITC Microbeads | Mouse |  | 130-048-701 | Miltenyi Biotec |
| Anti-APC Microbeads | Mouse |  | 130-090-855 | Miltenyi Biotec |
| Anti-PE Microbeads | Mouse |  | 130-048-801 | Miltenyi Biotec |
| Pax7 | Mouse |  |  | DSHB |
| Myosin heavy chain | Mouse | MF20 |  | DSHB |
| Anti-mouse IgG Alexa Fluor 488 | Goat |  | 115-545-003 | Jackson ImmunoResearch |
| Anti-rat IgG Alexa Fluor 488 | Goat |  | 112-545-003 | Jackson ImmunoResearch |
| Anti-rabbit IgG Alexa Fluor 488 | Goat |  | 111-545-003 | Jackson ImmunoResearch |

| **Negative in FGF-2-treated cells** | | | | | | | | |
| --- | --- | --- | --- | --- | --- | --- | --- | --- |
| CD1a | CD20 | CD45RA | CD84 | CD134 | CD196 | CD309 | Vβ8 | TRA-1-60 |
| CD1b | CD21 | CD45RB | CD85 | CD135 | CD197 | CD314 | CD326 | TRA-1-81 |
| CD1d | CD22 | CD45RO | CD86 | CD137 | CD205 | CDw327 | mlgM |  |
| CD2 | CD23 | CD48 | CD87 | CD137L | CD206 | CDw328 | mlgG1 |  |
| CD3 | CD25 | CD50 | CD88 | CD150 | CD209 | CD329 | mlgG2a |  |
| CD4 | CD27 | CD53 | CD89 | CD153 | CD220 | CD335 | mlgG2b |  |
| CD4v4 | CD28 | CD62E | CD94 | CD154 | CD226 | CD336 | mlgG3 |  |
| CD5 | CD31 | CD62L | CD100 | CD158a | CD229 | CD337 | CD104 |  |
| CD6 | CD32 | CD62P | CD102 | CD158b | CD231 | αβTCR | CD120b |  |
| CD7 | CD33 | CD64 | CD103 | CD161 | CD235a | CLIP | CD132 |  |
| CD8a | CD35 | CD66 | CD114 | CD162 | CD243 | CMRF-56 | CD210 |  |
| CD8b | CD36 | CD66b | CD116 | CD163 | CD244 | fMLP-R | CD212 |  |
| CD11a | CD37 | CD66f | CD121a | CD172b | CD255 | yδTCR | CD267 |  |
| CD11b | CD38 | CD69 | CD121b | CD177 | CD268 | HPC | CD294 |  |
| CD11c | CD39 | CD70 | CD122 | CD178 | CD273 | HLA-DQ | CLA |  |
| CD14 | CD41a | CD72 | CD123 | CD180 | CD274 | HLA-DR | ITGN-β7 |  |
| CD15s | CD41b | CD74 | CD124 | CD181 | CD278 | HLA-DP | rlgM |  |
| CD16 | CD42a | CD79b | CD126 | CD183 | CD279 | I.NKT | rlgG1 |  |
| CD18 | CD42b | CD80 | CD127 | CD193 | CD282 | NKB1 | rlgG2a |  |
| CD19 | CD43 | CD83 | CD128b | CD195 | CD305 | Vβ23 | rlgG2b |  |

A


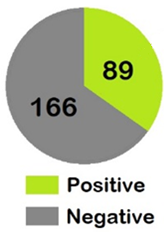

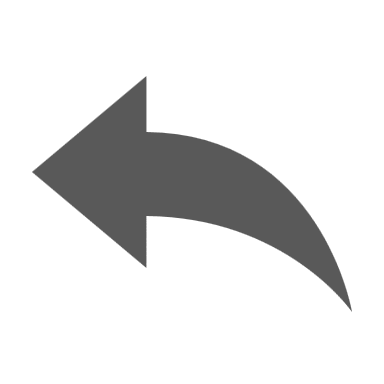


| **Expression level not different between FGF-2(+) and FGF-2(‒)** | | | | | | |
| --- | --- | --- | --- | --- | --- | --- |
| CD34 | CD59 | CD99 | CD119 | CD151 | CD275 | EGF-R |
| CD44 | CD63 | CD99R | CD130 | CD152 | CD321 | HLA-ABC |
| CD45 | CD71 | CD105 | CD138 | CD165 | CD331 | HLA-A2 |
| CD46 | CD75 | CD107a | CD140a | CD166 | CD332 | SSEA-4 |
| CD47 | CD81 | CD107b | CD140b | CD171 | CD333 | BLTR-1 |
| CD49c | CD90 | CD108 | CD141 | CD200 | CD334 | CMRF-44 |
| CD49e | CD91 | CD112 | CD142 | CD221 | CD340 | MIC A/B |
| CD51/61 | CDw93 | CD117 | CD144 | CD227 | β2-microglobulin | |
| CD58 | CD97 | CD118 | CD147 | Disialoganglioside GD2 | | |


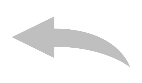
B
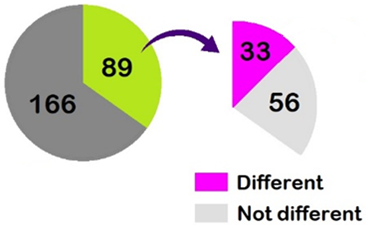


| **High in FGF-2(+)** | |
| --- | --- |
| CD29 | CD146 |
| CD56 | CD164 |
| CD57 | CD184 |
| CD77 | CD271 |
| CD120a | CD338 |

| **Low in FGF-2(+)** | | | | |
| --- | --- | --- | --- | --- |
| CD9  CD40 | CD26  CD54 | CD49d | CD73 | CD201 |
| CD10  CD49a | CD30  CD55 | CD49f | CD95 | SSEA-1 |
| CD13  CD49b | CD40 | CD54 | CD98 | SSEA-3 |
| CD15  CD49d | CD49a | CD55 | CD106 |  |
| CD24  CD49f | CD49b | CD61 | CD109 |  |

C
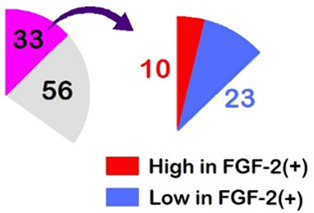


**Supplementary Figure S1. Surface marker profile of human iPSC-derived myogenic progenitors.** (A) 89 surface markers that were not detected in FGF-2-treated cells. (B) 56 surface markers that did not show noticeable different expression levels between FGF-2-treated and non-treated cells. (C) 10 surface markers that showed higher expression in FGF-2-treated cells and 23 that showed lower expression in FGF-2-treated cells.

**
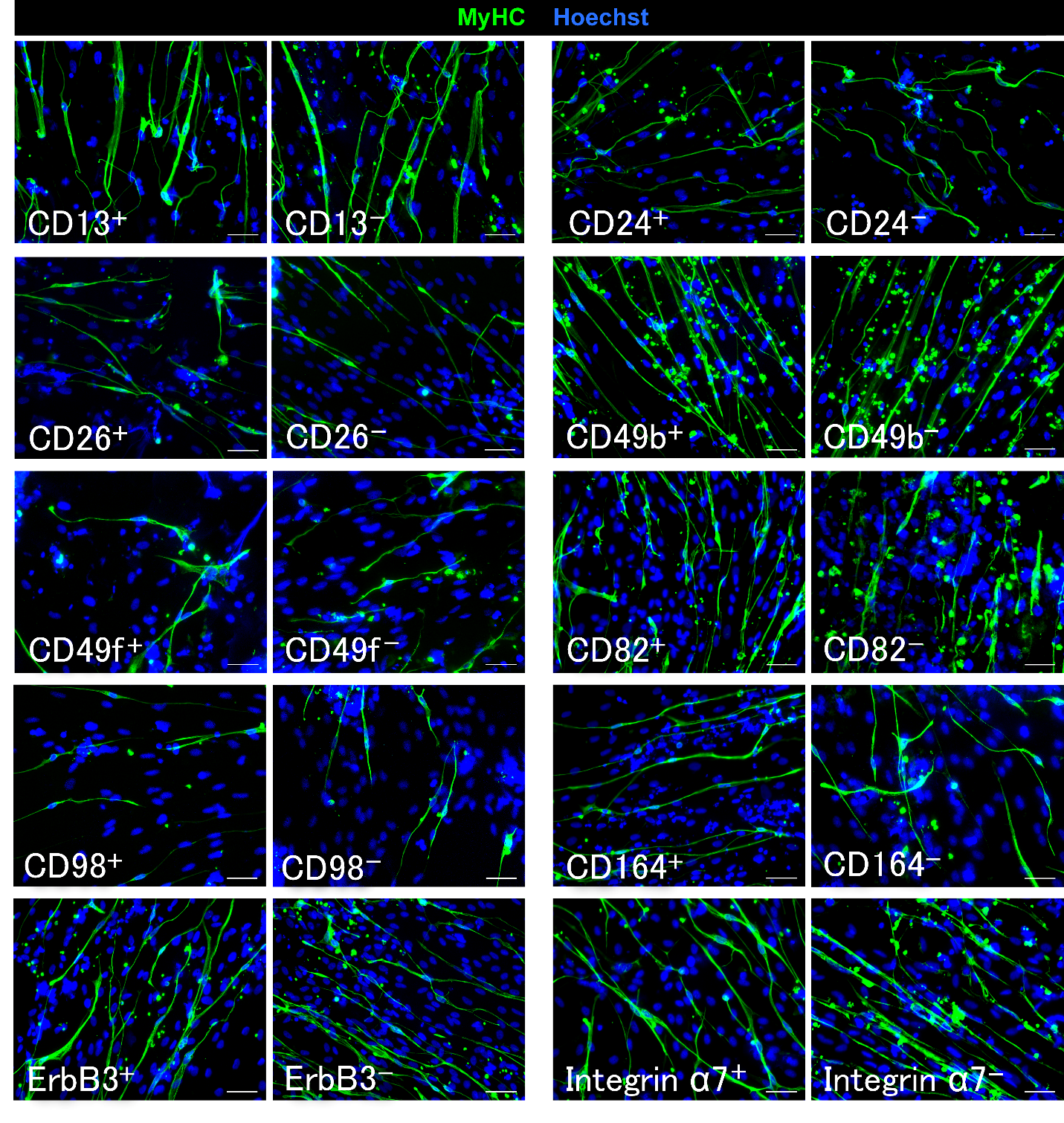
**

**Supplementary Figure S2. Surface markers that failed to enrich human iPSC-derived myogenic progenitors.** Visibly similar number of myotubes were found in both positive and negative fractions of human iPSC-derived EZ sphere cells sorted based on their expression of CD13, CD24, CD26, CD49b, CD49f, CD82, CD98, CD164, ErbB3 and Integrin α7, these markers were unable to enrich for myogenic progenitors. Scale bar = 20 µm.


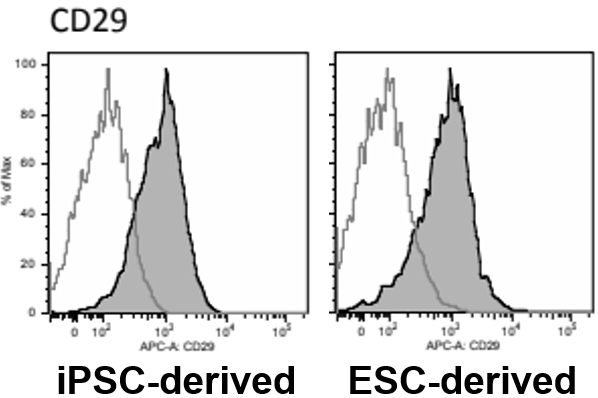

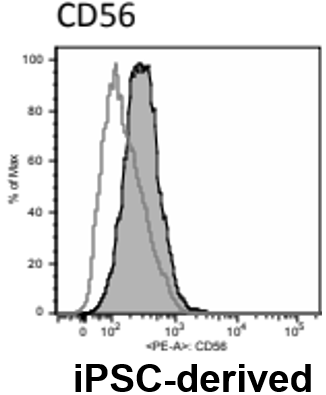

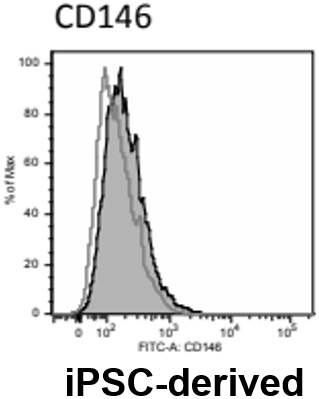

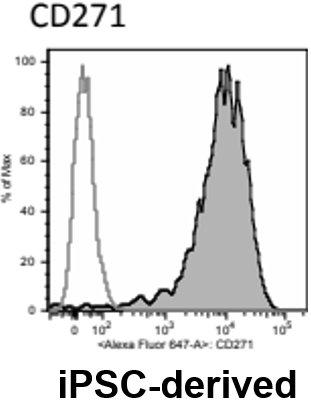

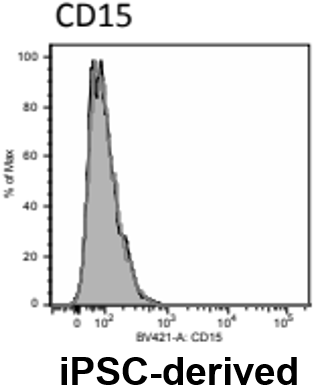

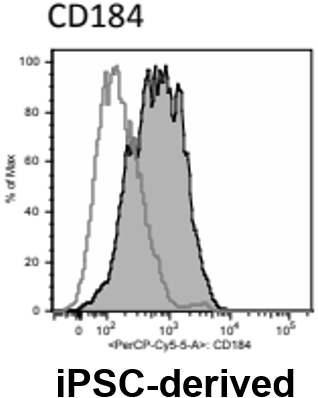


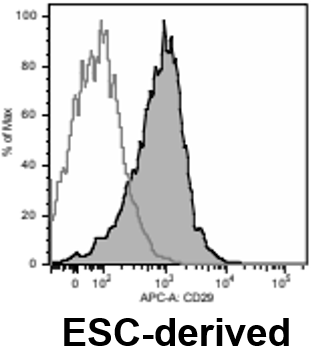

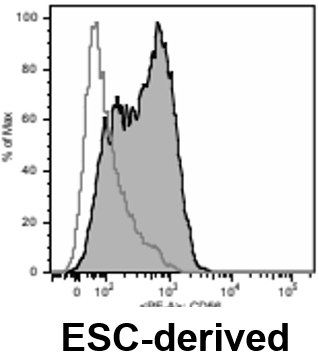

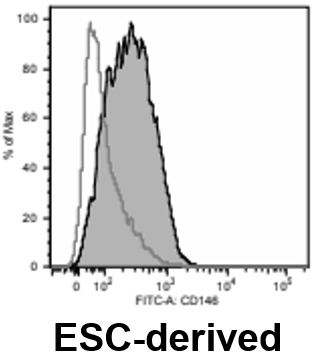

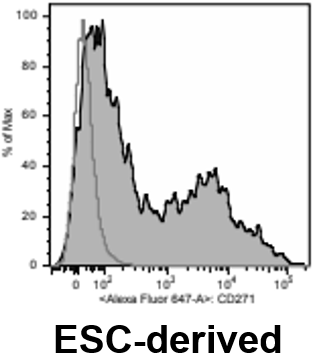

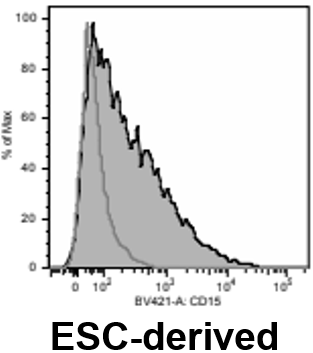

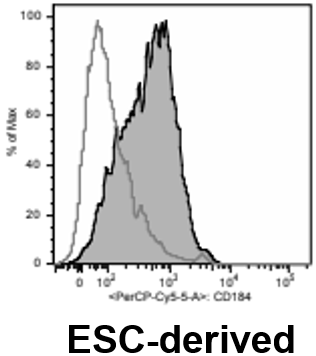


**Supplementary Figure S3. Surface marker profiling of human PSC-derived myogenic progenitors by flow cytometry.** Expression of positive markers CD29, CD56, CD146, CD271, negative marker CD15, and “no enrichment” marker CD184 (from left to right) in human iPSC-derived (top) and ESC-derived (bottom) FGF-2-treated EZ sphere cells.
